# Unidentified Recurrent Acute Compartment Syndrome of the Right Upper Limb

**DOI:** 10.7759/cureus.22033

**Published:** 2022-02-08

**Authors:** Ahmad Shiraz, Hira Bakhtiar, Ghazi Farman, Salman Khan, Nawal Rafiq

**Affiliations:** 1 General Surgery, Hayatabad Medical Complex (HMC), Peshawar, PAK; 2 Department of Community Medicine, Jinnah Medical College, Peshawar, PAK; 3 Accident and Emergency, Rehman Medical Institute, Peshawar, PAK; 4 Anesthesiology, Rehman Medical Institute, Peshawar, PAK; 5 Medicine and Surgery, Khyber Medical University, Peshawar, PAK

**Keywords:** osteofascial compartment, upper extremity, intracompartmental pressure, fasciotomy, acute compartment syndrome

## Abstract

Acute compartment syndrome (ACS) is a surgical emergency that requires urgent fasciotomy to prevent irreversible sequelae. Symptoms usually include intense pain, tenderness in the affected area, tingling or burning sensation, and in severe cases, numbness or weakness and limb amputation due to ischemia from compression of the blood vessels, respectively. This case report describes a 19-year-old female who presented with complaints of severe pain and swelling in her right forearm. On examination, no bite marks, blisters, or skin necrosis were noted except for several surgical scars from her previous surgeries for the same condition, i.e., compartment syndrome. Upon thorough examination, including relevant investigation and clinical judgment, she was diagnosed with acute compartment syndrome, for which she underwent fasciotomy. There was no basic underlying pathology found in her case, making it an unidentified case of acute compartment syndrome.

## Introduction

Acute compartment syndrome (ACS) is a surgical emergency that occurs when there is increased pressure within a closed osteofascial compartment, resulting in compression of surrounding tissues and local circulation. It is considered a surgical emergency when it is not treated in a timely manner; it can lead to ischemia, necrosis, infection, and amputation. Generally, acute compartment syndrome is considered a clinical diagnosis. However, intracompartmental pressure (ICP) and radiological scans can be used as a threshold to aid in diagnosis. ACS commonly involves the anterior compartment of the leg, but it can also be seen in the forearm, thigh, buttock, shoulder, hand, and foot [1-4]. Diagnosis is usually made on clinical judgment. However, clinical judgment alone has been proven to have inadequate diagnostic performance characteristics [5]. Usually, compartment syndrome is traumatic, but it can rarely present with no underlying etiology. Hence, this case is reported as idiopathic recurrent ACS of the right upper limb.

## Case presentation

A 19-year-old unmarried female from Afghanistan presented to us with no previous comorbidities with complaints of swelling in the right upper limb with severe deep and aching pain. She had a previous history of recurrent compartment syndrome since 2018, for which no underlying etiology has been found. Moreover, she had no other history of medical relevance and no preceding injury.

Upon examination, there was redness and swelling on the medial and anterior aspect of the right forearm, along with pain from passive touch and decreased two-point discrimination. On deep palpation, the involved compartment was firm and wooden hard, which was suggestive of compartment syndrome. Moreover, no neurological deficit or paresthesia was noted.

**Figure 1:**
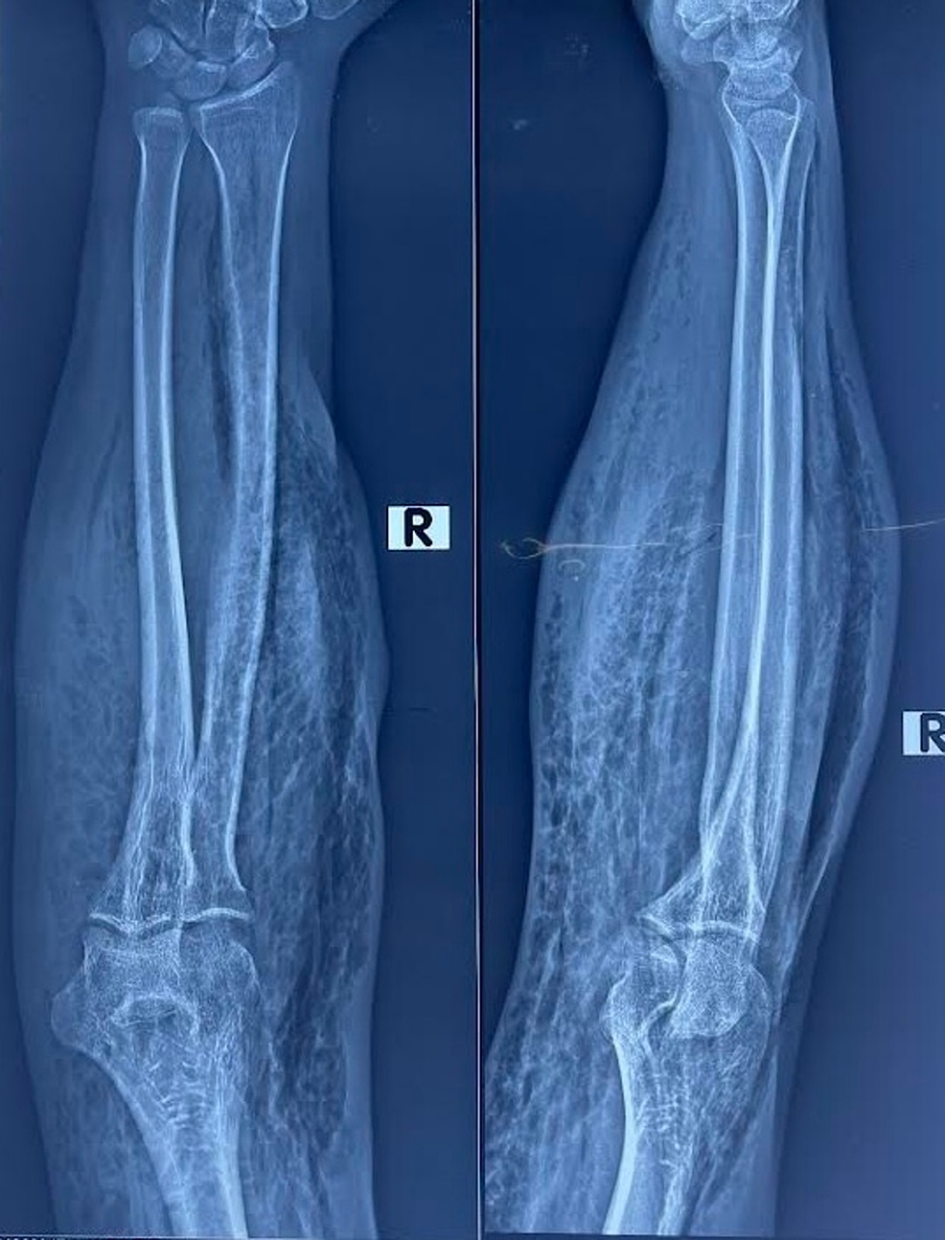
X-ray of right forearm X-ray right forearm showing subcutaneous emphysema of the soft tissues of the upper forearm. No fracture, bone deformity, or any trauma is noted.

**Figure 2:**
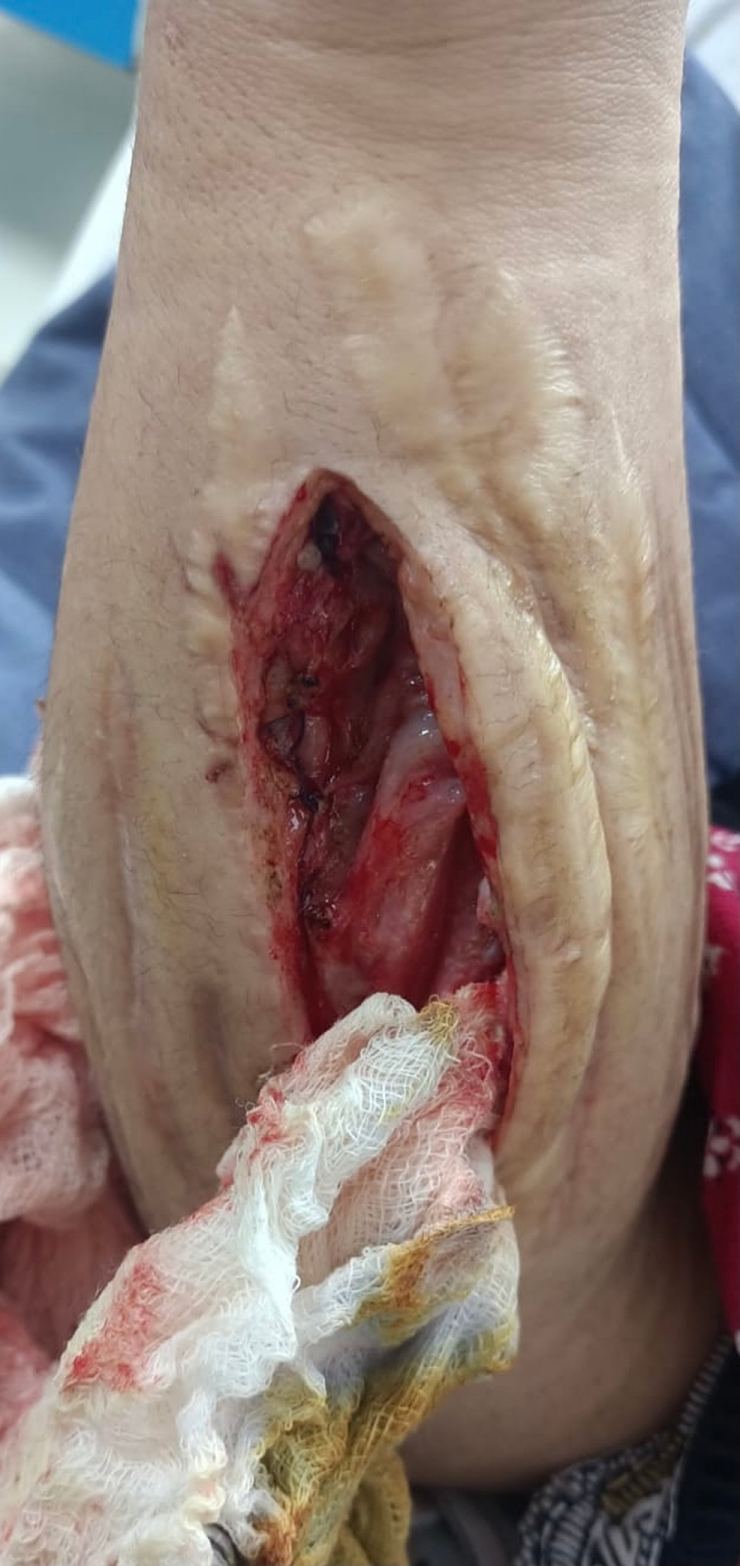
Post fasciotomy picture showing several longitudinal scars from previous surgeries performed on the right forearm

Routine blood tests were normal except for hemoglobin (9.2g/dL) and blood urea (15mg/dL). An X-ray of the right forearm revealed emphysema in the subcutaneous and deep soft tissue planes (Figure 1). The patient was vitally stable, and acute compartment syndrome was suspected on clinical judgment. She was prepared for the fasciotomy of the forearm with per-operative findings of air in the subcutaneous tissue and swelling in the right forearm, along with several longitudinal surgical scars from her previous surgeries for the same condition (Figure 2). As it was a case of unidentified recurrent ACS, this time during surgery, a tissue sample (muscle) was taken for biopsy, culture, and sensitivity. Postoperative recovery of the patient was uneventful, and hence the patient was discharged on the third postoperative day on 400mg Nezkil (linezolid) tablets and Danzen® Ds tablets to prevent postoperative infections.

On follow-up, the culture and sensitivity showed no growth from the specimen after 24 hours of aerobic incubation at 35°C, and muscle biopsy revealed necrosis, and mixed inflammatory infiltrates. Special stains for fungus were also negative, and there was no evidence of malignancy on biopsy.

**Figure 3:**
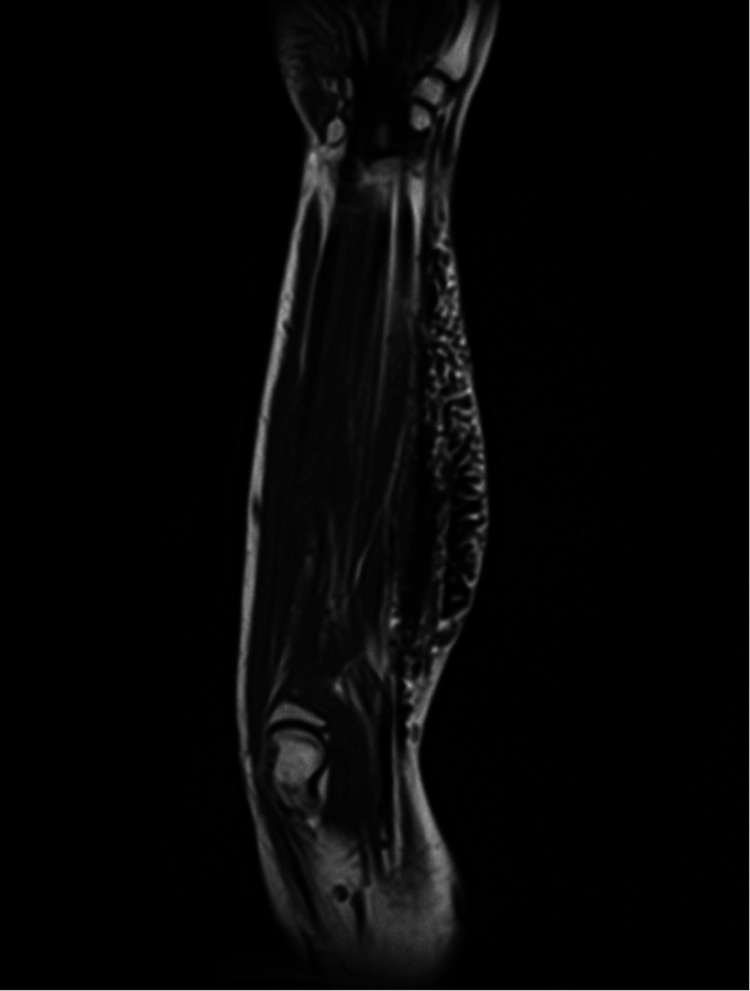
MRI of right forearm MRI of the right forearm shows subcutaneous emphysema and changes of cellulitis involving the anterolateral aspect of the forearm with extensive edema signals in proximal intermuscular facial planes without any definitive evidence of osseous or muscular involvement.

After two months, she presented to us in the emergency department with a similar presentation of ACS along with crepitus in the similar area for which she underwent emergency fasciotomy. We started a thorough investigation to rule out the underlying etiology of ACS. A multidisciplinary team was formed to discuss the case summary of the patient, and all previous investigations and scans were reviewed and discussed to find out any cause of recurrent compartment syndrome. A previous MRI of the right forearm report (2019) showed no fracture or dislocation. Muscles of the extensor and flexor were normally visualized with no abnormality. However, subcutaneous soft tissue swelling with multiple serpiginous T1 T2/short tau inversion recovery (STIR) hypointense signal abnormality areas in the anterior and lateral aspect of the forearm corresponding to the region of swelling representing surgical emphysema with a combination of changes of cellulitis were noted (Figure 3).

**Figure 4:**
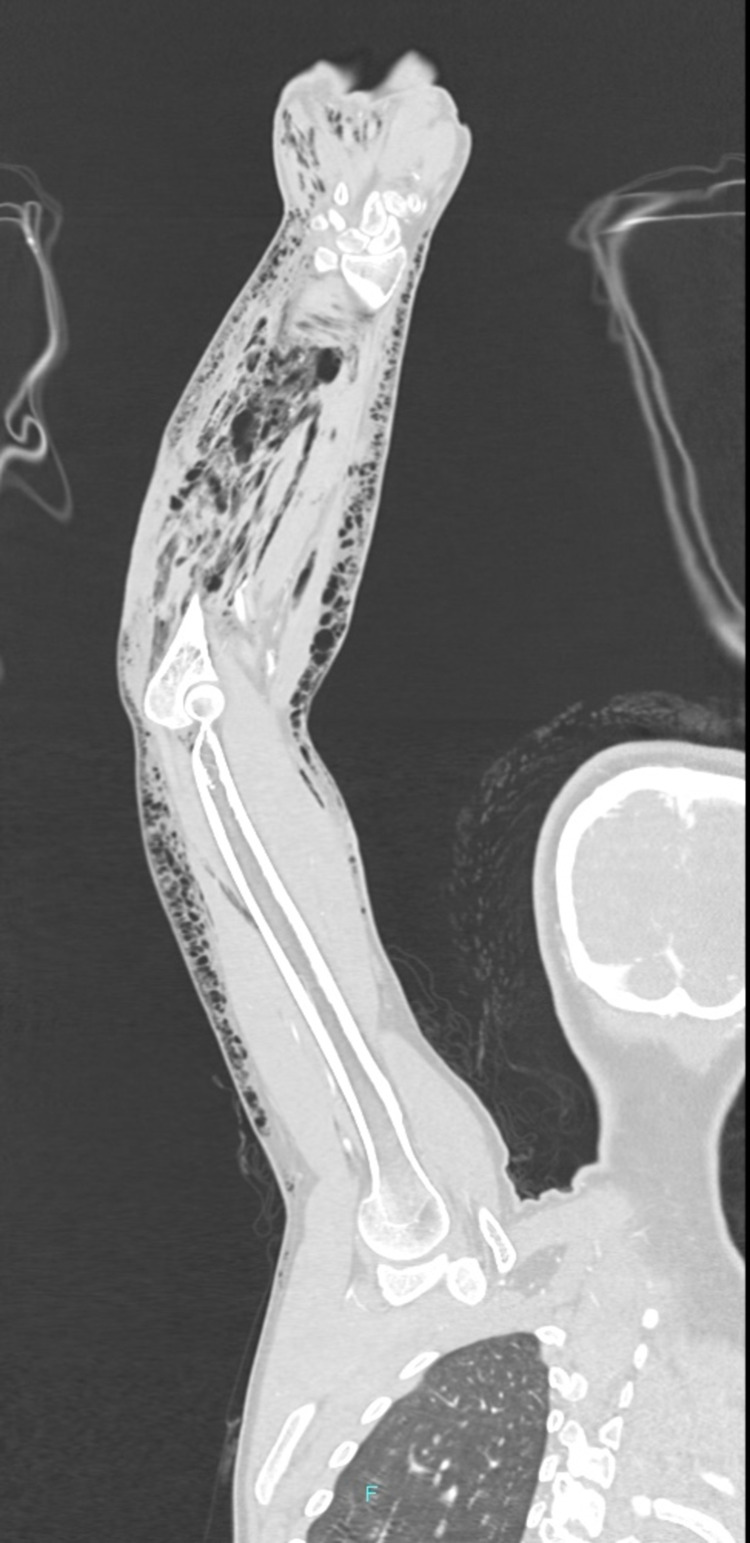
CTA of the right upper limb CTA of the right upper limb shows extensive emphysema and edema in the entire right upper limb in subcutaneous and deep soft tissues. Right brachiocephalic trunk, subclavian, axillary, brachial, radial, and ulnar arteries are grossly patent with no stenosis or malformation. CTA - computed tomography angiography

Tissue microbiology reports from previous surgeries showed the growth of two organisms: *Pseudomonas aeruginosa* and *Acinetobacter baumannii* isolated after 24 hrs of incubation, suggesting the hospital-acquired infection for which she was commenced on intravenous meropenem after antimicrobial sensitivity. However, blood cultures revealed no growth. Finally, a decision was made to do computed tomography angiography (CTA) for the upper limb to rule out any arterial defect. The CTA reported extensive emphysema in the entire right upper limb from the axilla through the hand, involving subcutaneous and deep tissues as well as all muscles and intermuscular planes. The right brachiocephalic trunk, subclavian, axillary, brachial, radial, and ulnar arteries were grossly patent with no stenosis or malformation. (Figure 4).

After insignificant findings, we decided to discharge the patient with a diagnosis of idiopathic recurrent ACS. She was also educated about her condition and to report to us if she were to develop any similar episodes in the future.

## Discussion

An acute painful limb is a common but difficult presentation, with variable medical diagnosis. A detailed history, clinical examination, and basic investigations usually help narrow the differential diagnosis [6]. For the clinical diagnosis of acute compartment syndrome, six cardinal signs are typically taken into consideration, including pain, pulselessness and pallor, paresthesia and paralysis, and poikilothermia. The diagnosis is mainly clinical. However, not all the symptoms or signs may be present initially [7,8]. Idiopathic acute compartment syndrome is uncommon, which makes diagnosing ACS more difficult [9,10].

Our case was interesting as our patient presented with the classical picture of acute compartment syndrome despite the lack of an obvious insult or mechanism predisposing to this surgical emergency, labeling it as a case of unidentified ACS.

An uncommon cause for the development of acute compartment syndrome has been linked to athletes taking creatinine supplementation and individuals who are engaged in intensive physical exercise [11]. This was not the case with our patient. Another diagnosis of exclusion is acute exertional compartment syndrome, which is secondary to elevated intra-compartmental pressures in the absence of trauma usually seen among athletes [12]. Our patient had no obvious risk factors, intrinsic or acquired, that predisposed her to develop acute compartment syndrome, making her situation an idiopathic compartment syndrome. This is a very uncommon phenomenon, rarely reported in the medical literature, with only a few cases reported to date [13,14].

Acute compartment syndrome is an important surgical emergency that should not be missed. Classical presentation and clinical knowledge should help in the diagnosis, even in its unusual forms of presentation. Our patient did present to us over a regular period of 1.5-2 months with unidentified recurrent ACS. To date, she has undergone multiple fasciotomies with no obvious cause.

## Conclusions

Acute compartment syndrome is a surgical emergency and should not be missed. However, idiopathic acute compartment syndrome is rare. Timely diagnosis and clinical judgment followed by prompt fasciotomy can be a limb-saving measure. Our case highlights the development of recurrent ACS with no underlying cause. Moreover, it also warrants further study to establish the cause of her recurrent acute compartment syndrome.
